# *Rheum officinale* Baill.: chemical characterization and *in-vitro* biological activities

**DOI:** 10.3389/fchem.2025.1661223

**Published:** 2025-12-10

**Authors:** Fatima Kerroum, Aicha Atoui, Latifa Khattabi, Mustafa Abdullah Yilmaz, Oguz Cakir, Ayomide Victor Atoki, Mohammed Messaoudi

**Affiliations:** 1 Biotechnology Research Center-C.R.Bt, Constantine, Algeria; 2 Laboratory of the Development and Valorization of Plant Genetic Resource, Faculty of Sciences, Brothers Mentouri University, Constantine, Algeria; 3 Department of Analytical Chemistry, Faculty of Pharmacy, Dicle University, Diyarbakir, Türkiye; 4 Science and Technology Research and Application Center, Dicle University, Diyarbakir, Türkiye; 5 Department of Nutrition and Dietetics, Faculty of Health Sciences, Dicle University, Diyarbakir, Türkiye; 6 Department of Biochemistry, Kampala International University, Ishaka, Uganda; 7 Laboratory for Research on Bioactive Products and Biomass Valorization, Department of Chemistry, ENS Kouba, Algiers, Algeria

**Keywords:** *Rheum officinale*, antimicrobial activity, enzyme inhibition, antioxidant, phytochemical profile

## Abstract

Medicinal plants constitute a valuable natural resource of bioactive phytochemicals, which are increasingly studied for their therapeutic potential and broad applications in the pharmaceutical, nutraceutical, and cosmetic fields. *Rheum officinale*, a medicinal rhubarb species, is appreciated for the presence of biologically active compounds with therapeutic relevance. This work analyses the chemical composition, including the phytochemical profile, and pharmacological activities of *Rheum officinale* Baill. stems in Algeria. The plant extract was analyzed for its notable antioxidant capacity using various assays, including DPPH, ABTS, β-carotene bleaching, ferric and also cupric reducing power, and metal chelation. The inhibitory potential against cholinesterase and α-amylase was assessed through specific enzymatic assays. LC–ESI-MS/MS assessment highlighted the phytochemical profile within the extract, with quinic acid identified as the major component. Antimicrobial potential against *P*. *aeruginosa, S*. *aureus, E*. *coli, E. faecalis, and C*. *albicans* was confirmed *via* agar diffusion and inhibition zone (C) tests. The extract demonstrated potent antioxidant activity, with radical scavenging IC_50_ values less potent than reference antioxidants such as BHT and α-tocopherol (IC_50_ = 0.42 ± 1.43 μg/mL). Total phenol and flavonoid content were quantified using Folin-Ciocalteu and AlCl_3_ methods, yielding high values (373.10 ± 0.055 mg GAE/g and 38.012 ± 0.05 mg QE/g, respectively). Enzyme inhibition assays demonstrated significant activity against key enzymes related to Alzheimer’s disease (IC_50_: 28.14 ± 2.22; 73.71 ± 1.48 μg/m) and diabetes (IC_50_: 36.21 ± 0.56 μg/m). The extract also exhibited antimicrobial effects. Given its bioactive potential, *Rheum officinale* presents promising opportunities for therapeutic product development, supporting the pharmaceutical industry.

## 1 Introduction

The discovery and development of novel pharmaceuticals are significantly influenced by natural products, particularly those originating from plants (Krajewska et al., 2024; Yener et al., 2020). Natural products are valuable sources of leads for pharmaceutical research due to their extensive biological activities and structural diversity (Demir et al., 2024; Oncu et al., 2024; Yilmaz et al., 2024). Consequently, traditional medicine, which has been practiced for centuries, is founded on plants that possess potent medicinal properties, including antioxidant, enzyme inhibitory, and antimicrobial activity (Findik et al., 2024).

The *Rheum* genus includes approximately 60 species of robust herbaceous species (Xiang et al., 2020). Perennial rhubarb has eatable stalks, it has hard stems with a characteristic woody appearance, long leaves, and clusters of small, wind-pollinated flowers (Lee et al., 2017). *Rheum officinale* Baill., belonging to the Polygonaceae family, is widely cultivated in TCM (Xiong et al., 2019). Known in China as the “ruler or king of herbs,” rhubarb has been used for more than 2,000 years in traditional medicine due to its wide range of pharmacological properties (Wang et al., 2018). Its name in Arabic is “Raound, الرواند.” Rhubarb is a source of biologically active ingredients necessary for the treatment and prevention of lifestyle related diseases due to its laxative, diuretic, antidiabetic, antibacterial, hemostatic, anti-inflammatory, antiviral, immunosuppressive and antitumor properties (Huang et al., 2019; Jintao et al., 2018; Shang et al., 2019; Stompor–gorący, 2021). It contains several valuable bioactive phytochemicals such as anthraquinones, dianthrones, stilbenes, and flavonoids (Xie et al., 2020; Zhang et al., 2024), which contribute to improving the health status of humans and animals. It also has a high content of dietary fiber (Goel et al., 1999). It should be noted that the fresh shoots and stems of *Rheum officinale* are used for the treatment of many diseases, but rhubarb leaves can be poisonous, as they contain a high concentration of oxalates, unlike stems and petioles (Clementi and Misiti, 2010). *R. officinale* has been shown to possess antioxidant (Emen Tanrikut et al., 2013; Kalisz et al., 2020), antimicrobial (Alaadin et al., 2007), and antihyperglycemic properties (Kasabri et al., 2011).

In North America, Europe, and several Middle Eastern regions, some *Rheum* species are traditionally used in sweet, fruit-based preparations. Their value in the diet stems from their richness in bioactive constituents and dietary fiber. In particular, *Rheum rhabarbarum* is widely used in culinary applications for the preparation of desserts, cakes, mousses, juices, wines, and fruit teas (Dai et al., 2022). In Algeria, people use *R. officinale* stems as food prepared similarly to spinach dishes, valued for its beneficial effects against indigestion, stomach pain, haemorrhoids, and diarrhea. In our research, the phytochemical profile of the hydromethanolic extract derived from local *R. officinale* stems was analyzed using LC-ESI-MS/MS. Furthermore, its biological potential was assessed through evaluations of radical-scavenging ability, antimicrobial effectiveness, and inhibition of key metabolic enzymes.

## 2 Materials and methods

### 2.1 Reagents and chemicals

All reagents, solvents, and standards used throughout the experiments were supplied by Sigma-Aldrich (French).

### 2.2 Extraction of plant material

Rhubarb stems were collected in January 2020 from the Babur Mountains (Easten Algeria) and authenticated at the Botanic Authentication Laboratory of Ahmed Ben Bella University (Oran1). The stems were first rinsed, air-dried and finely powdered. A portion of 10 g of the ground plant material was macerated in 100 mL of 80% methanol, filtered and evaporated with (Buchi, Germany) to obtain the hydromethanolic extract of rhubarb stems (HMERS).

### 2.3 Phytochemical analysis of HMERS

The total phenolic content of HMERS was determined using the Folin–Ciocalteu method (Singleton and Rossi, 1965) with slight modifications (Müller et al., 2010). The mixture was freshly prepared by combining 100 μL of the diluted Folin–Ciocalteu reactive solution (1:10) and sodium carbonate solution at 75 g/L with 20 μL of the extract. The prepared reaction mixture was subjected to incubation for 2 h and then the reading was taken at 740 nm. The amount of total phenolic was determined and presented as gallic acid (mg GAE per g of extract).

For flavonoid quantification, the procedure performed using the method of the aluminum chloride colorimetric method described by (Djeridane et al., 2006). HMERS solution was prepared (0.125 mg/mL) and combined with 2% AlCl_3_ in methanol. Spectrophotometric readings were taken at 430 nm. The calibration curve was established using quercetin standards (5–50 μg/mL), and results were reported as mg QE per g (DW).

### 2.4 *In vitro* evaluations of antioxidant activity

#### 2.4.1 The β-carotene bleaching test

The antioxidant activity of HMERS was evaluated using β-carotene linoleic acid model system (Benahmed et al., 2021). The β-carotene stock solution was prepared in chloroform (0.5 mg/mL), mixed with Tween 40 (200 mg) and linoleic acid (25 μL), and then evaporated under vacuum. 100 mL of H_2_0_2_ was introduced with vigorous shaking to obtain a stable emulsion. Aliquots (4 mL) were combined with different extract concentrations, and the values were read at 470 nm (0 h and 2 h of incubation at 50 °C). A control was used for correction (without β-carotene) and inhibition rate was determined according to the next equation:

$$\mathrm{PI}\%=\left[1‐\left(\left(\frac{\mathrm{As}\;\left(t0‐t120\right)}{\;\mathrm{Ac}\;\left(t0‐t120\right)}\right)\right)\times \;100\right]$$

Where, *As* is the absorbance values of the sample, whereas *Ac* refers to the control absorbance.

#### 2.4.2 DPPH test

DPPH free radical scavenging test of HMERS was determined by the assay described by Blois (1958). For the assay, a DPPH solution was freshly prepared (0.1 mM in methanol) and mixed with sample (160 μL: 40 μL) in different dilutions. After an incubation period of 30 min at room temperature in the dark, the absorbance at 517 nm was recorded using a 96-well microplate reader (EnSpire Multimode Plate Reader, PerkinElmer). The inhibitory potency was represented by IC_50_ values. Inhibition rate (%) was obtained using the formula below:

$$IP\;\left(\%\right)=\frac{Ab\;control-Ab\;sample}{Ab\;control}x100$$

Where: Ab = absorbance.

#### 2.4.3 Reduction of copper cation test

The cupric reducing antioxidant capacity of HMERS was assed following the procedure adopted by Apak et al. (2004). To perform the assay, 40 µL of the extract was mixed with 60 µL of ammonium acetate (CH_3_COONH_4_), 50 µL of neocupronin, and 50 µL of copper (II) chloride dihydrate (CuCl_2_, 2H_2_O) in a suitable reaction vessel. After gentle homogenization, the blend was maintained 1 h under incubation, and the measured absorbance was recorded at 450 nm.

#### 2.4.4 ABTS cation decolorization test

The spectrophotometric test of ABTS^+^ scavenging ability was assessed as initiated by Re et al. (1999). For this assay, a stock solution of ABTS^+^ (160 μL) was combined with sample (40 μL) in methanol at varying dilutions. After incubation of the prepared mixture, the optical density was measured at 734 nm and the relative activity (%) was estimated according to the equation:

$$\mathrm{Inhibition}\;effect\;\left(\%\right)=\frac{\left(A\;\text{control}\;‐\;A\;\text{sample}\right)}{A\;\text{control}}\times \;100$$

where A: is the absorbance.

#### 2.4.5 Ferric cation reduction test

The reducing power of the extract was measured (Oyaizu, 1986). Different concentrations of the sample extract (10 μL each) were prepared and 0.2 M phosphate buffer (pH 6.6) containing 1% potassium ferricyanide were introduced into the sample. After reaction with trichloroacetic acid and ferric chloride, the spectrophotometric measurement was taken at 700 nm and the values were presented as the concentration (μg mL^−1^) required to achieve an absorbance of 0.5 (A_0.5_).

#### 2.4.6 O-phenanthroline test

The assay was carried out by combining 30 µL of 0.5% O-phenanthroline, 50 µL of FeCl_3_ (0.2%), 10 µL of sample extract, and 110 µL of methanol at various concentrations. The obtained mixture was maintained for 20 min at 30 °C and its reading absorbance was subsequently assessed at 510 nm. The percentage of inhibition was then calculated relative to an appropriate control (Khattabi et al., 2022).

### 2.5 Enzymatic inhibition tests

#### 2.5.1 The *in vitro* anti-Alzheimer potential of HMERS

The anticholinesterase potential was performed by mixing the extract or galantamine (10 mL) with 20 μL portion of enzyme solution (6.85 × 10^−3^ U for BChE or 5.32 × 10^−3^ U for AChE) and 150 µL of phosphate buffer (100 mM, pH 8.0). After incubation, 10 μL of the substrate solution (acetyl or butyrylthiocholine) and an equal volume of DTNB (0.5 mM) were subsequently added to the first reaction, and measurement of absorbance was carried out at 412 nm. The percentage of enzyme inhibition was calculated using the formula:

$$\text{Inhibition }\left(\%\right)=\left[\left(E\;‐\;S\right)/E\right]\times 100$$

where E is the enzyme activity in the absence of the test sample, and S is the enzyme activity in its presence (Ellman et al., 1961).

#### 2.5.2 *In vitro* anti-diabetic activity of *R. officinale* by alpha amylase inhibition assay

α-amylase inhibitory activity was performed using iodine/potassium iodide (IKI) method (Zengin et al., 2014), with some modifications. The assay involved incubating varying concentrations of the sample (extract or acarbose) with α-amylase (1 U) for (10 min; 37 °C), then added starch solution at 0.1% concentration, HCl and IKI. Sample absorbance was quantified at 630 nm and the inhibition percentage of the enzyme was resolute as:

$$\left(\%\right)\mathrm{Inhibition}=\frac{\left(A\;\text{control}\;‐\;A\;\text{control}\;\text{blanc}\right)\;–\;\left(A\;\text{sample}\;–\;A\;\text{sample}\;\text{blanc}\right)}{\left(A\;\text{control}\;–\;A\;\text{control}\;\text{blanc}\right)}\times 100$$

Where (A) are the absorbance values.

### 2.6 Antimicrobial potential

Antimicrobial effect of HMERS was assessed against several strains, among them *E*. *coli* ATCC 8739, *S*. *aureus* ATCC 6538, *E*. *faecalis* ATCC 49452, *P*. *s aeruginosa* ATCC 27853, and the fungal strain *C*. *albicans* ATCC 90026, obtained from the Microbiology Laboratory of Tamanrasset University. Determination of the minimum inhibitory concentration (MIC) was performed according to the broth microdilution technique. Twofold serial dilutions of the extract (20–104 μg/mL) were prepared in DMSO (≤2%), which did not show any noticeable effect on microbial growth. An equal volume (100 µL) of the extract dilution and the microbial inoculum (10^6^ CFU/mL) was added to each well. Negative (broth only) and positive (microorganism without extract) controls were included. The MIC was defined after being incubated for 24 h at 37 °C as the minimum concentration of the extract that prevented visible microbial growth. All experiments were carried out in three replicates.

### 2.7 Mass spectrometer and chromatograph conditions

Authors used a Shimadzu-Nexera UHPLC system (SIL-30AC autosampler, CTO-10ASvp oven, LC-30AD pumps, DGU-20A3R degasser) and a Shimadzu LCMS-8040 triple quadrupole mass spectrometer to measure the amounts of 53 phytochemicals (Supplementary Table S1) (Yilmaz, 2020). Samples were separated on an Agilent Poroshell 120 EC-C18 column (150 × 2.1 mm, 2.7 μm) at 40 °C. Mobile phases were water (5 mM ammonium formate, 0.1% formic acid) as A and methanol with the same additives as B. The gradient progressed from 20% to 100% B over 0–25 min, held at 100% B until 35 min, then returned to 20% B by 45 min. Flow rate was 0.5 mL/min, injection volume 5 μL. Mass detection used electrospray ionization in positive and negative modes, with LabSolutions software for data processing. Quantification employed MRM with optimized precursor–product ion transitions. Ion source settings were: drying gas 15 L/min, nebulizing gas 3 L/min, interface 350 °C, desolvation line 250 °C, heat block 400 °C. Method validation parameters are listed in Supplementary Table S1.

### 2.8 Data analysis

The experimental data were subjected to one-way analysis of variance (ANOVA). Mean comparisons were carried out using Duncan’s multiple range test, with statistical significance considered at p < 0.05.

## 3 Results

As shown in Table 1, the TPC, TFC, and CTA assays revealed that the extract has considerable potential quantity of phenolic/flavonoid compounds and condensed tannins with IC50 value (373.10 ± 0.055 μg GAE/mg, 78.05 ± 0.004 μg QE/mg and 43.012 ± 0.05 μg CE/mg) of extract, respectively.

**TABLE 1:** Total phenolics content in *R. officinale* extracts.

Extract	Total phenolic (μg GAE mg^−1^ E)	Total flavonoids (μg QE mg^−1^ E)	Condensed tannins (μg CE mg^−1^ E)
HMERS	373.10 ± 0.055	78.05 ± 0.004	43.012 ± 0.05

TPC, total phenolic compounds; TFC, total flavonoids; CTA, condensed tannins. Data are presented as mean ± standard deviation from three independent replicates.

Tables 2 and 3 summarize the findings of the *in vitro* antioxidant and anti-enzymatic assays. Regarding the antioxidant performance of the extract, most of the assays revealed IC_50_ and A_0_._5_ values comparable to the reference standards, with statistically significant effects (P < 0.05). Notably, in the ABTS assay, the extract exhibited a stronger response, reaching high significance (P < 0.01; IC_50_ = 0.42 ± 1.43 μg/mL). Likewise, for the enzyme inhibition tests, the IC_50_ values for cholinesterase inhibition (AChE and BCHE) were close to those of the reference compound, galantamine, which served as the positive control since it is clinically applied in the management of mild Alzheimer’s disease. In contrast, the anti-α-amylase test provided the most effective inhibition, as reflected by the lowest IC_50_ value (36.21 ± 0.56 μg/mL) lower than that of acarbose, indicating strong α-amylase inhibitory potential.

**TABLE 2:** Antioxidant properties of HMERS presented as IC_50_ and A_0_._5_ values (μg/mL).

Extract/Standard	A_0.5_			IC_50_		
	CUPRAC	FRAP	Phenanthroline	β-carotene	DPPH	ABTS
HMERS	18.47 ± 0.34^a^	16.21 ± 0.56^a^	10.84 ± 0.88^a^	18.61 ± 0.70^a^	13.36 ± 0.78^a^	0.42 ± 1.43^a^
BHT*	5.35 ± 0.71^b^	nt	0.93 ± 0.07^b^	0.91 ± 0.01^b^	6.14 ± 0.41^b^	1.29 ± 0.30^b^
BHA*	8.97 ± 3.94^c^	nt	2.24 ± 0.17^c^	1.05 ± 0.03^c^	12.99 ± 0.4^c^	1.81 ± 0.10^c^
Ascorbic acid*	nt	6.77 ± 1.15^b^	nt	nt	nt	nt
αTocopherolb*	nt	34.93 ± 2.3^c^	nt	nt	13.02 ± 5.1^c^	nt

* Standard compounds. nt, not tested. The IC_50_ and A0.5 values were derived from linear regression analysis and are presented as mean ± standard deviation from three independent replicates. Values with different superscript letters (a, b, c) in the same parameter were significantly different (P < 0.05).

**TABLE 3:** Anti-enzymatic activity of HMERS represented as IC_50_ (μg/mL).

Stems extract	Anti- AChE	Anti-BChE	Anti-alpha amelase
HMERS	28.14 ± 2.22^a^	73.71 ± 1.48^a^	36.21 ± 0.56^a^
Galantamine*	6.27 ± 1.15^b^	34.75 ± 1.99^b^	-
Acarbose*	-	-	3650.93 ± 10.70^b^

* Standard compounds. -, no activity. The IC_50_ values values were derived from linear regression analysis and reported as mean ± standard deviation from three replicates. Values with distinct superscripts letters (a, b) in a column denote significant variation (p < 0.05).

**FIGURE 1:**
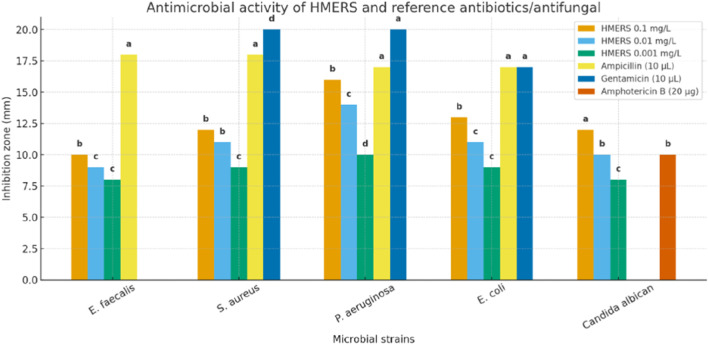
Antimicrobial activity of EMERS.

The antimicrobial activity result of our extract at different concentrations also the positive controls (ampicillin, gentamicin and amphotericin B) are showed in Table 4. The funding indicate that HMERS exhibited a significant antimicrobial effect (*p* < 0.05) against the different microorganisms studied, although the degree of activity varied comparing to the standard antibiotics (Figure 1).

**TABLE 4:** HMERS’s antibacterial activity (mean ± SD. n = 5).

Microbial strains	HMERS (mg L^−1^)			Ampicillin (μL)	Gentamicin (μL)	Amphotericin B (µg)
	0.1	0.01	0.001	10	10	20
*E. faecalis*	10 ± 0.01^b^	9 ± 0.00^c^	8 ± 0.00^c^	18 ± 0.01^a^	-	-
*S. aureus*	12 ± 0.00^b^	11 ± 0.01^b^	9 ± 0.00^c^	18 ± 0.01^a^	20 ± 0.01^e^	-
*P. aeruginosa*	16 ± 0.02^b^	14 ± 0.0^c^	10 ± 0.02^d^	17 ± 0.01^a^	20 ± 0.01^a^	-
*E. coli.*	13 ± 0.01^b^	11 ± 0.01^c^	9 ± 0.01^c^	17 ± 0.01^a^	17 ± 0.01^a^	-
*Candida albicans*	12 ± 0.00^a^	10 ± 0.00^b^	8 ± 0.02^c^	-	-	10 ± 0.02^b^

Diameter of disc used is equal to 6 mm. Values are reported as mean ± standard deviation from three replicates, those with different letters (a, b, c, d) above the bars denote significant differences (p < 0.05) and values with the same letters are not significantly different (p > 0.05) among treatments within each microbial strain. Comparisons were not made between different microorganisms.

Diameter of disc used is equal to 6 mm. Different letters above the bars denote significant differences (p < 0.05) among treatments within each microbial strain. Comparisons were not made between different microorganisms.

**FIGURE 2:**
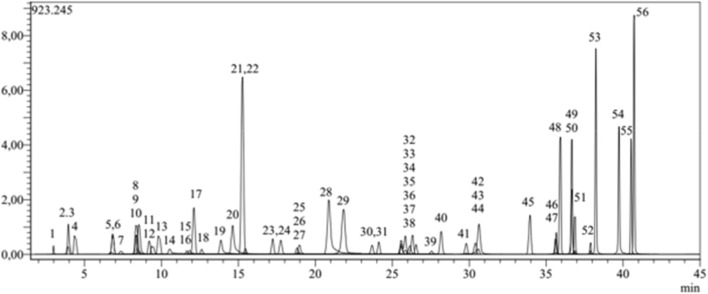
Total ion chromatogram of standard phenolic compounds analysed by the developed LC–MS/MS method (Karagecili et al., 2023).

**FIGURE 3:**
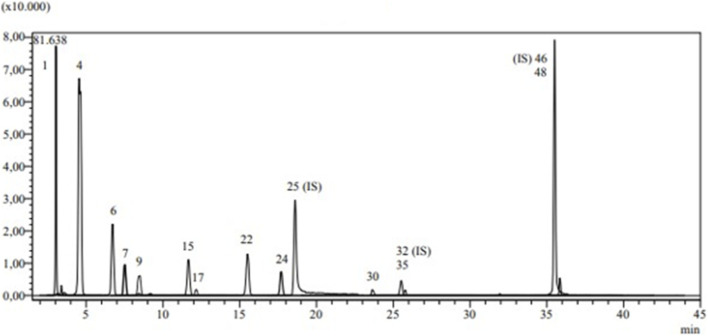
Chemical profile of EMERS using LC–MS/MS.

The identity of the medicinal plant was established using a rigorously validated LC-MS/MS methodology. Among the 53 phytochemicals included in the developed method, several phenolic (fifty) and non-phenolic (tree) were detected in our extract. The LC–MS/MS total ion current (TIC) chromatograms of the 53 standards phytochemicals and HMERS exposed in Figures 2, 3, respectively). In addition, the quantitative LC–MS/MS data are presented in (Table 5). The investigation revealed that phenolic acids represented the major group of polyphenols in this plant, with quinic, gallic, chlorogenic, protocatechuic, caffeic, p-coumaric, and tannic acids identified; quinic acid being the predominant coumpond in HMERS.

**TABLE 5:** Quantitative screening of phytochemicals in HMERS.

No	Analytes	R.T.	M.I. (m/z)	F.I. (m/z)	Amount (mg/g)	No	Analytes	R.T.	M.I. (m/z)	F.I. (m/z)	Amount (mg/g)
1	**Quinic acid**	3.0	190.8	93.0	**5.059**	29	Salicylic acid	21.8	137.2	65.0	N.D.
2	Fumaric aid	3.9	115.2	40.9	N.D.	30	Cyranoside	23.7	447.0	284.0	0.082
3	Aconitic acid	4.0	172.8	129.0	N.D.	31	Miquelianin	24.1	477.0	150.9	N.D.
4	**Gallic acid**	4.4	168.8	79.0	**2.601**	32	Rutin-D3-IS	25.5	612.2	304.1	N.A.
5	Epigallocatechin	6.7	304.8	219.0	N.D.	33	Rutin	25.6	608.9	301.0	N.D.
6	Protocatechuic acid	6.8	152.8	108.0	0.764	34	Isoquercitrin	25.6	463.0	271.0	N.D.
7	Catechin	7.4	288.8	203.1	0.355	35	Hesperidin	25.8	611.2	449.0	0.036
8	Gentisic acid	8.3	152.8	109.0	N.D.	36	o-Coumaric acid	26.1	162.8	93.0	N.D.
9	Chlorogenic acid	8.4	353.0	85.0	0.201	37	Genistin	26.3	431.0	239.0	N.D.
10	Protocatechuicaldehyde	8.5	137.2	92.0	N.D.	38	Rosmarinic acid	26.6	359.0	197.0	N.D.
11	Tannic acid	9.2	182.8	78.0	0.266	39	Ellagic acid	27.6	301.0	284.0	N.D.
12	Epigallocatechin gallate	9.4	457.0	305.1	N.D.	40	Cosmosiin	28.2	431.0	269.0	N.D.
13	Cynarin	9.8	515.0	191.0	N.D.	41	Quercitrin	29.8	447.0	301.0	N.D.
14	4-OH Benzoic acid	10.5	137.2	65.0	N.D.	42	Astragalin	30.4	447.0	255.0	N.D.
15	**Epicatechin**	11.6	289.0	203.0	**3.734**	43	Nicotiflorin	30.6	592.9	255.0/284.0	N.D.
16	Vanilic acid	11.8	166.8	108.0	N.D.	44	Fisetin	30.6	285.0	163.0	N.D.
17	Caffeic acid	12.1	179.0	134.0	0.055	45	Daidzein	34.0	253.0	223.0	N.D.
18	Syringic acid	12.6	196.8	166.9	N.D.	46	Quercetin-D3-IS	35.6	304.0	275.9	N.A.
19	Vanillin	13.9	153.1	125.0	N.D.	47	Quercetin	35.7	301.0	272.9	N.D.
20	Syringic aldehyde	14.6	181.0	151.1	N.D.	48	Naringenin	35.9	270.9	119.0	0.022
21	Daidzin	15.2	417.1	199.0	N.D.	49	Hesperetin	36.7	301.0	136.0/286.0	N.D.
22	**Epicatechin gallate**	15.5	441.0	289.0	**1.499**	50	Luteolin	36.7	284.8	151.0/175.0	N.D.
23	Piceid	17.2	391.0	135/106.9	N.D.	51	Genistein	36.9	269.0	135.0	N.D.
24	p-Coumaric acid	17.8	163.0	93.0	0.067	52	Kaempferol	37.9	285.0	239.0	N.D.
25	Ferulic acid-D3-IS^h^	18.8	196.2	152.1	N.A.	53	Apigenin	38.2	268.8	151.0/149.0	N.D.
26	Ferulic acid	18.8	192.8	149.0	N.D.	54	Amentoflavone	39.7	537.0	417.0	N.D.
27	Sinapic acid	18.9	222.8	193.0	N.D.	55	Chrysin	40.5	252.8	145.0/119.0	N.D.
28	Coumarin	20.9	146.9	103.1	N.D.	56	Acacetin	40.7	283.0	239.0	N.D.

RT, retention time; M.I., molecular ion; F.I., fragment ion; N.A., not applicable; N.D., not detected.

Bold values indicate the major compounds in the extract.

**FIGURE 4:**
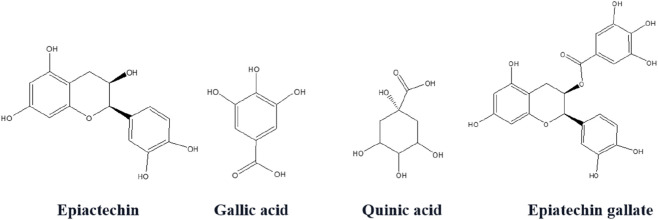
Chemical structures of the major bioactive compounds detected in HMERS.

According to the Table 5, *R. officinale* stems exhibited a notably high quinic acid content (5.059 analyte/g extract), highlighting this species as a significant natural source of quinic acid, followed by epicatechin, gallic acid and epicatechin gallate (Figure 4).

## 4 Discussion

Our survey aimed at profiling the phytochemical content of *Rheum officinale* stems cultivated in Algeria using a hydromethanolic extract and to assess their inhibitory effect on cholinesterase enzymes (AChE and BChE), and digestive carbohydrate enzymes, mainly α-amylase, along with their antioxidant and antimicrobial potentials. The LC–MS/MS profiling of HMERS identified a set of bioactive phytochemicals, even though, numesrous molecules were not detected (N.D.) or present in trace quantities. The profile highlights both phenolic acids and flavonoids, which are well-known as key contributors to antioxidant, antimicrobial, enzyme-inhibitory, and protective biological activities. Quinic acid, present at the highest concentration (5.059 mg/g), was the predominant compound in HMERS. As an important metabolite with antioxidant, anticancer, antidiabetic, hepatoprotective, and neuroprotective properties (Li et al., 2024; Samimi et al., 2021), its high presence in the rhubarb extract likely explains the observed antioxidant activity and suggests a role in attenuating oxidative stress-related conditions, including Alzheimer’s disease and metabolic dysfunctions. According to our LC-MS/MS analyses acquired data, study of Benali et al. (2024) identified that quinic acid as the most abundant phenolic compound in *Rheum officinale* leaf extracts, with a concentration of 129.686 mg/g. This finding suggests that quinic acid may contribute to the antioxidant and anti-inflammatory properties of the plant. The other most abundant constituent within the extract was gallic acid, with 2.601 mg/g. As a potent antioxidant, it also exhibits anti-inflammatory, anticancer, and antimicrobial properties (Kahkeshani et al., 2019; Keyvani-Ghamsari et al., 2023). Its high concentration likely underlies part of the extract’s strong radical-scavenging activity. Followed by epicatechin (3.734 mg/g) and epicatechin gallate (1.499 mg/g), these flavan-3-ols are well-documented antioxidants having cardiovascular, antidiabetic, and neuroprotective benefits (Márquez Campos et al., 2020). They are known also to modulate carbohydrate metabolism and neurotransmitter degradation, supporting the inhibitory activity against α-amylase and cholinesterases. Their presence in stems of rhubarb further reinforces the plant’s pharmacological value in both traditional and modern medicinal applications. Similar study reported that rhubarb extracts (*Rheum officinale* leaf) contain flavan-3-ols at concentrations ranging from 86.57 to 195.98 mg per 100 g of dry matter, depending on the variety and harvest period (Dai et al., 2024). Protocatechuic acid (0.764 mg/g) and catechin (0.355 mg/g), both contribute to antioxidant and antimicrobial effects. Protocatechuic acid, particularly, is associated with hepatoprotective and nephroprotective activities, consistent with rhubarb’s traditional use against renal disorders. Other minor compounds detected in trace amounts (<0.1 mg/g), such as, caffeic acid (0.055 mg/g), p-coumaric acid (0.067 mg/g), cyranoside (0.082 mg/g), hesperidin (0.036 mg/g), and naringenin (0.022 mg/g). Although present in low concentrations, these molecules are bioactive flavonoids and phenolic acids with anti-inflammatory, antimicrobial, and metabolic regulatory functions. Their synergistic interactions with major compounds may enhance the pharmacological profile of the extract (Kakkar and Bais, 2014; Kassab et al., 2022; Khattabi et al., 2022).

Species of the genus *Rheum*, including *R. officinale*, are widely recognized for their therapeutic potential. Rich in phenolic acids, flavonoids and anthraquinones. This species possesses a broad spectrum of biological activities, including antioxidant, anti-inflammatory, antimicrobial, anticancer, antidiabetic, hepatoprotective, and laxative effects. Their bioactive compounds contribute to protection against oxidative stress, modulation of metabolic disorders, and support of digestive and liver health, which underlies their long-standing use in traditional medicine (Xiang et al., 2020; Yang et al., 2024). In our extract, several common phytochemicals, such as anthraquinones, rutin, quercetin, syringic acid, ferulic acid, and kaempferol, were undetected. Their absence may reflect species-specific phytochemistry, methanol extraction selectivity, or a lower accumulation in stems comparing to roots or leaves.

According to our results, rhubarb stems exhibit significant antimicrobial action, particularly against *S. aureus,* which is highly sensitive to its compounds such us quinic acid. Studies suggest that rhubarb exerts these effects by altering membrane permeability, inhibiting protein synthesis, and disrupting respiratory metabolism (Lingqing et al., 2021; Xiang et al., 2017). The study by Li et al. (2014) focused on the antibacterial activity of quinic acid against *Staphylococcus aureus* by demonstrating that quinic acid reduces membrane fluidity and interferes with the normal function of the bacterial cell membrane. The researchers found that quinic acid, along with chlorogenic acid, possessed wide-ranging antibacterial effects. Other molecules also extracted from different species of *Rheum* include anthraquinones and its derivatives, such as emodin, rhein, and aloe-emodin, show remarkable antibacterial effects *in vitro* against various strains, such as *S. aureus, Lactobacillus*, and *E. coli* (Ji et al., 2017; Jiang et al., 2019; Stompor–Gorący, 2021).

Starch is the main source of digestible carbohydrates in the human food and the major contributor to postprandial glucose levels. Its enzymatic hydrolysis is carried out by α-amylase and α-glucosidase, the main catalysts involved in carbohydrate digestion. Inhibiting these enzymes is a well-established therapeutic strategy to control hyperglycemia by limiting glucose absorption (Melakhessou et al., 2021). In parallel, Cholinesterases, namely (AChE) acetylcholinesterase and (BChE) butyrylcholinesterase, act as key enzymes involved in neurotransmission. Dysregulation of their activity is strongly associated with Alzheimer’s disease. In this study, rhubarb stem extract showed significant *in vitro* inhibition of AChE, BChE, and α-amylase, consistent with the reported neuroprotective and antidiabetic potential of its constituents. Our results suggest that the combined presence of phenolic acids (e.g., gallic, protocatechuic, caffeic, and p-coumaric acids) and flavan-3-ols (epicatechin, catechin) contributes to the observed enzyme inhibition, thereby supporting the therapeutic relevance of the (HMERS). Previous studies have similarly documented the effective inhibition of these enzymes by rhubarb-derived preparations. Moretheless (Xie et al., 2022), highlighted that anthraquinones, flavanols and their polymers, as well as phenolic acids such as gallic acid, represent core bioactive constituents of rhubarb responsible for its multifunctional pharmacological properties. Previous studies demonstrated that anthraquinones such as emodin, chrysophanol, rhein, and danthron have shown therapeutic potential in Alzheimer’s disease models (Cao et al., 2017; Li et al., 2019). Notably, rhein-derived hybrids inhibited key enzymes (AChE, BChE, BACE-1), reduced Aβ aggregation *in vitro,* and mitigated oxidative stress and tau pathology (Pérez-Areales et al., 2017). Similarly, tacrine–rhein hybrids further combined anti-amyloid and metal-chelating activities with fewer side effects (Li et al., 2014). Furthermore, rhubarb has long been used in traditional medicine for the management of diabetic nephropathy (DN) and is frequently combined with conventional drugs for enhanced efficacy (Cao et al., 2017; Huang et al., 2023). Clinical investigation have demonstrated that treatment with rhubarb-based compounds significantly improves biochemical markers including serum creatinine, blood urea nitrogen, albumin, and fasting plasma glucose. The nephroprotective effects of rhubarb are ascribed to its ability to reduce urinary protein excretion, regulate lipid metabolism, improve renal function, and modulate key molecular markers. These actions help suppress renal inflammation and fibrosis, thereby slowing the progression of DN (Gao and Nan, 2022; Zhang et al., 2023). In summary, our findings provide compelling evidence that rhubarb (*R. officinale*) contains bioactive compounds, including phenolic acids and flavan-3-ols, which collectively contribute to its antioxidant, antibacterial, antidiabetic, and neuroprotective activities. These results not only support traditional uses of rhubarb but also highlight its potential as a valuable reservoir of multifunctional molecules with therapeutic relevance.

## 5 Conclusion

Rhubarb is recognized as one of the most valuable medicinal species, extensively applied in ancestral healing systems due to its therapeutic efficacy. The findings of this study demonstrate that *Rheum officinale* stems possess a remarkable profile of bioactive compounds, primarily phenolics and flavonoids, which contribute to their potent antioxidant, enzyme-inhibitory, and antimicrobial potential. These biological properties suggest that *R. officinale* could serve as a promising natural source for developing nutraceuticals and therapeutic agents targeting oxidative stress, neurodegenerative disorders, and metabolic diseases.

To fully harness its pharmacological potential, subsequent research should emphasize the purification and structural characterization of its bioactive molecules, combined with *in vivo* evaluations of their therapeutic effectiveness and safety. These additional investigations would contribute to a clearer understanding of the mechanisms underlying its biological effects and support its possible translation into clinical applications.

## Data Availability

The original contributions presented in the study are included in the article/Supplementary Material, further inquiries can be directed to the corresponding author.
